# Relationship between 1,25-Dihydroxyvitamin D and Body Composition in Middle-Aged Sedentary Adults: The FIT-AGEING Study

**DOI:** 10.3390/nu11112567

**Published:** 2019-10-24

**Authors:** Alejandro De-la-O, Lucas Jurado-Fasoli, Manuel J. Castillo, Luis Gracia-Marco, Ángel Gutierrez, Francisco J. Amaro-Gahete

**Affiliations:** 1 EFFECTS 262 Research Group, Department of Physiology, Faculty of Medicine, University of Granada, 18071 Granada, Spain; juradofasoli@ugr.es (L.J.-F.); gutierre@ugr.es (Á.G.); mcgarzon@ugr.es (M.J.C.); 2 PROFITH “PROmoting FITness and Health Through Physical Activity” Research Group, Sport and Health University Research Institute (iMUDS), Department of Physical Education and Sports, Faculty of Sport Sciences, University of Granada, 18071 Granada, Spain; lgracia@ugr.es

**Keywords:** vitamin D, calcitriol, body mass index, lean mass, fat mass, bone mineral density

## Abstract

Vitamin D deficiency is a worldwide health problem that, in addition to its well-known negative effects on musculoskeletal health, has been related to a wide range of acute and chronic age-related diseases. However, little is known about the association of body composition with the active, hormonal form of vitamin D, 1,25-dihydroxyvitamin D plasma levels (1,25(OH)_2_D). Therefore, the aim of this study was to investigate the association of 1,25(OH)_2_D with body composition including lean and fat body mass as well as bone mineral density (BMD) in middle-aged sedentary adults. A total of 73 (39 women) middle-aged sedentary adults (53.7 ± 5.1 years old) participated in the current study. We measured weight and height, and we used dual energy X-ray absorptiometry to measure lean body mass, fat body mass and BMD. Body mass index (BMI), lean mass index (LMI), and fat mass index (FMI) were calculated. 1,25(OH)_2_D was measured using a DiaSorin Liaison® immunochemiluminometric analyzer. The results showed a negative association of 1,25(OH)_2_D with BMI, LMI and BMD (β = −0.274, R^2^ = 0.075, *p* = 0.019; β = −0.268, R^2^ = 0.072, *p* = 0.022; and β = −0.325, R^2^ = 0.105, *p* = 0.005, respectively), which persisted after controlling for age and sex. No significant differences in 1,25(OH)_2_D across body weight status were observed after controlling for the same covariates. In summary, our results suggest that 1,25(OH)_2_D could be negatively associated with BMI, LMI and BMD whereas no association was found with FMI in middle-aged sedentary adults.

## 1. Introduction

As the world´s population ages, the prevalence of chronic diseases increases, particularly over the last decades, becoming one of the great challenges that society faces [1,2]. Abnormalities in body composition such as a decrease of lean body mass and/or bone mineral density (BMD) or an increment in fat body mass are powerful predictors of morbidity and mortality risk as well as overall quality of life [3]. Epidemiologic studies indicate that these body composition changes are closely related to obesity, sarcopenia and/or osteoporosis in the elderly population [3,4]. However, these chronic diseases are progressive and initiate at a younger age [5,6]. Their high prevalence and concomitant health risk make them a particularly relevant worldwide public health problem and a social and economic burden [7,8,9].

Vitamin D is a fat-soluble vitamin essential for normal homeostasis of calcium and phosphorus, as well as for bone health [10] and preventing falls [11] and fractures [12]. Globally, vitamin D deficiency has been considered as a major public health problem affecting not only musculoskeletal health but also a wide range of several age-related chronic diseases [13]. 25-hydroxyvitamin D (25(OH)D) is the most commonly used biomarker when evaluating the relationship of vitamin D status with health-related outcomes [14,15,16,17]. However, 25(OH)D is thought to be largely inactive since it requires to be metabolized in the kidney by the enzyme 25-hydroxyvitamin D-1α-hydroxylase to be active. Therefore, 1,25-dihydroxyvitamin D (1,25(OH)_2_D), also known as calcitriol, is responsible for most, if not all, of its biological effects [18,19]. The association between 1,25(OH)_2_D and body composition parameters has been hard to establish for several reasons: (i) previously there was not a reliable and sensitive assay for calcitriol [20,21] and (ii) 25(OH)D has a higher concentration and longer half-life than 1,25(OH)_2_D, thus requiring less sample volume for reliable measurements [22].

Consequently, epidemiological data are scarce, and a review of the scientific literature found no large studies examining the relationship between 1,25(OH)_2_D and body composition outcomes. There is some evidence of the existence of an inverse association of 1,25(OH)_2_D with body mass index (BMI) and fat body mass [23,24], however these data are conflicting with other studies [25,26,27,28]. In contrast, data are scarce on the potential relationship between 1,25(OH)_2_D and lean body mass. Two recent studies found a significant association between low 1,25(OH)_2_D and low lean body mass [29,30], which is highly dependent on the individual’s age [29]. Similarly, controversial findings have been discovered regarding the role of 1,25(OH)_2_D in bone health. Previous cross-sectional studies have reported an inverse association between 1,25(OH)_2_D and BMD [31,32,33], whereas other studies found that 1,25(OH)_2_D was unrelated to bone mineral content [34], bone loss [33,35] or hip fracture risk [36,37]. 

There are limited data on the study of body composition parameters in relation to 1,25(OH)_2_D status in middle-aged adults. Thus, understanding whether 1,25(OH)_2_D is associated with body composition parameters in this population is of clinical interest since, as previously established, the interventions to delay or reverse body composition related diseases are preferable when individuals are still relatively young and healthy [38,39].

Therefore, the aim of this study was to investigate the association of 1,25(OH)_2_D with body composition including lean and fat body mass as well as BMD in middle-aged sedentary adults. 

## 2. Materials and Methods

### 2.1. Study Design and Participants

The present cross-sectional study was conducted under the framework of the FIT-AGEING study (clinicaltrial.gov: ID: NCT03334357) [40]. The Ethics Committee on Human Research of the Regional Government of Andalucía approved the rationale, design, and methodology of the study [0838-N-2017] and all participants signed written informed consent in accordance with the Declaration of Helsinki (last revision guidelines, 2013).

Seventy-three middle-aged sedentary adults were recruited via electronic media, social networks and leaflets. The inclusion criteria were as follows: (i) to be sedentary (i.e., less than 20 min of physical activity on less than 3 days/week), (ii) not to have had greater body weight changes than 3 kg in the past 3 months, (iii) to be aged between 45 and 65 years old, (iv) not to be a smoker, (v) to be taking no long-term medication, (vi) not to be pregnant and (vii) not to suffer from any chronic cardiometabolic disease. 

All tests were performed during September–October 2016/17 at the Sport and Health University Research Institute (iMUDS, Granada, Spain) and at the “Campus de la Salud” Hospital (Granada, Spain). 

### 2.2. Anthropometric Parameters and Body Composition Assessment

A pre-validated Seca model 799 scale and stadiometer (Seca, Hamburg, Germany) was used to measure body weight and height with light clothing and without shoes. BMI was subsequently calculated as weight (kg)/height (m^2^). Body composition outcomes were determined by a dual-energy X-ray absorptiometry scanner (Discovery Wi, Hologic, Inc., Bedford, MA, USA) obtaining lean body mass in kg, fat body mass in kg, and BMD in g/cm^2^. A spine phantom quality check scan was conducted on each study day. A whole-body scan was performed considering all manufacturer´s guidelines (i.e., the positioning of participants, the analysis of results and the quality controls among others). The APEX 4.0.2. software was used to draw an automatic delineation of anatomic regions. The lean mass index (LMI) was calculated as lean body mass (kg)/body height (m^2^). Similarly, we calculated the fat mass index (FMI) as fat body mass (kg)/body height (m^2^). Fat mass was also expressed as a percentage of the total body mass. The participants were categorized into three groups on the basis of BMI levels: (i) normal weight (BMI ≥ 18.5 and <25 kg/m^2^), (ii) overweight (BMI ≥ 25 and <30 kg/m^2^), and (iii) obese (BMI ≥ 30 kg/m^2^).

### 2.3. Dietary Intake Assessment

We performed a total of three 24-hour dietary recalls collected on non-consecutive days (one weekend day included). This validated method is able to determine the energy intake to within 8–10% of the current energy intake [41]. The interviews were meal sequence-based, in which a detailed description of the food consumed by the participants was recorded. The 24-hour dietary recalls were collected by an experimented and qualified research dietitian (L.J.-F.), using a photograph guide to improve the quality of the information provided on portion sizes of food and assisting participants in the estimation of the consumed food quantity [42]. The software EvalFINUT^®^ updated with data from USDA (U.S. Department of Agriculture) and BEDCA (“Base de Datos Española de Composición de Alimentos”) was used to calculate energy and micronutrient (i.e., vitamin D, calcium and phosphorus) intake derived from the 24-hour recalls.

### 2.4. Physical Activity Assessment

Physical activity levels were objectively assessed with a wrist-worn accelerometer (ActiGraph GT3X+, Pensacola, FL, United States) for 7 consecutive days (24 hours/day) [40]. The sampling frequency was previously set at 100 Hz to store raw accelerations [43]. The ActiLife v.6.13.3 software (ActiGraph, Pensacola, FL, United States) and the GGIR package (v.1.5-12^2^) in R (v.3.1.2^3^) were used to process these files [44,45]. The participants came to the laboratory and specific information about how to wear the accelerometer was given. They were also reminded to remove it only during water-based activities such as swimming or bathing. Only the participants who wore the accelerometer for ≥16 hours/day for 4 days (including 1 weekend day) were included in the analysis.

### 2.5. Blood Samples Assessment

A 10 mL peripheral blood sample was taken from the antecubital vein after overnight fasting. It was collected using the Vacutainer SST system (Becton Dickinson, Plymouth, UK) in ethylenediamine tetra-acetic acid-containing tubes. Blood samples were centrifuged at four thousand revolution per minute for seven minutes at 4 °C and stored at −80 °C. Plasma levels of 1,25(OH)_2_D were measured using a DiaSorin Liaison® immunochemiluminometric analyzer (DiaSorin Ltd, Wokingham, Berkshire, UK) and expressed in pg/mL. 

### 2.6. Statistical Analysis

Data were checked for normality with the use of distribution plots (i.e., visual check of histograms, and Q-Q plots) and the Shapiro-Wilk test. The descriptive parameters were reported as mean and standard deviation. 

Differences between sexes were examined using an independent samples T test. Given that no interaction for sex was observed (*p* > 0.05), data are presented for men and women together. Simple linear regression models were built to test the association of 1,25(OH)_2_D and body composition outcomes (i.e., BMI, LMI, FMI, and BMD). We also performed multiple linear regression models to analyze these associations controlling for age (Model 1), sex (Model 2), and age and sex (Model 3). Additionally, we also adjusted these models for total energy, vitamin D, calcium, phosphorus intake and/or physical activity levels (i.e., light, moderate-vigorous and total physical activity). To test whether 1,25(OH)_2_D was different across body weight status (i.e., normal-weight, overweight and obese individuals), an analysis of variance (ANOVA) was conducted. Moreover, we performed an analysis of covariance to test the differences of 1,25(OH)_2_D across weight status adjusting for age and sex. 

Data were analyzed with the use of the Statistical Package for Social Sciences (SPSS, v. 22.0, IBM SPSS Statistics, IBM Corporation, Armonk, NY, USA). Graphical plots were built using the GraphPad Prism 5 (GraphPad Software, San Diego, CA, USA). The level of significance was fixed at <0.05.

## 3. Results

Table 1 shows the descriptive parameters of our study participants by sex. No significant differences in 1,25(OH)_2_D were observed between men and women (*p* = 0.576). 

**Figure 1:**
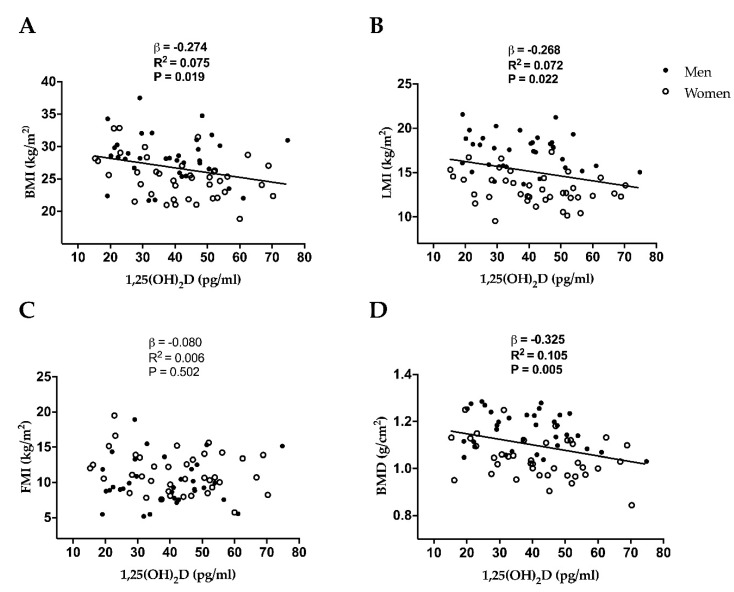
Simple linear regression graphs between 1,25-Dihydroxyvitamin D (1,25(OH)_2_D) and body mass index (BMI) (**A**), lean mass index (LMI) (**B**), fat mass index (FMI) (**C**), and bone mineral density (BMD) (**D**) in middle-aged sedentary adults. β (standardized regression coefficient), R^2^, and P from a simple linear regression analysis.

Figure 1 shows the associations between 1,25(OH)_2_D and body composition related parameters. There was a significant negative association of 1,25(OH)_2_D with BMI (β = −0.274, R^2^ = 0.075, *p* = 0.019, Figure 1A), LMI (β = −0.268, R^2^ = 0.072, *p* = 0.022, Figure 1B) and BMD (β = −0.325, R^2^ = 0.105, *p* = 0.005, Figure 1D), which persisted after including age, sex, and age and sex in the model (all *p* ≤ 0.042, Table 2). 1,25(OH)_2_D was not significantly associated with FMI (β = −0.080, R^2^ = 0.006, *p* = 0.502; Figure 1C), which did not change adjusting for age, sex, and age and sex (all *p* ≥ 0.35, Table 2). The results remained unchanged after further adjusting for total energy, vitamin D, calcium, phosphorus intake and/or physical activity levels (i.e., light, moderate-vigorous and total physical activity) (data not shown, all *p* > 0.1).

**Figure 2:**
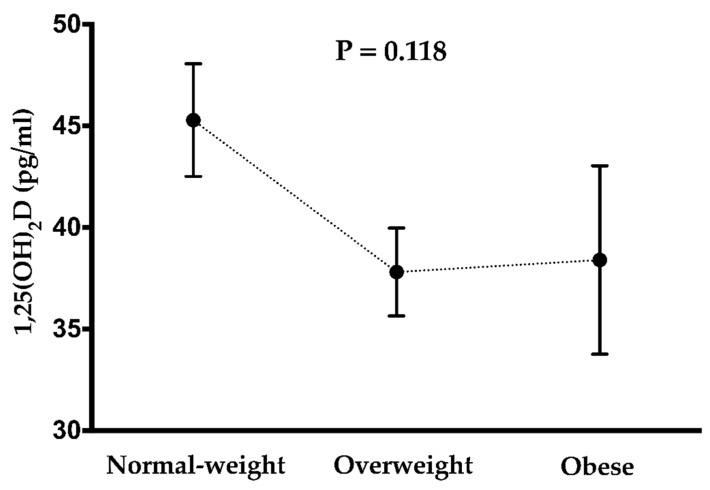
1,25-Dihydroxyvitamin D (1,25(OH)_2_D) by body weight status categories in middle-aged adults. Values are presented as means and standard error. *p* value obtained from the analysis of the variance to compare 1,25(OH)_2_D across weight status (normal-weight, over-weight, and obese).

ANOVA revealed no significant differences in 1,25(OH)_2_D across body weight status (45.3 ± 13.3 pg/mL in normal-weight, 37.8 ± 13.1 pg/mL in overweight and 38.4 ± 16.7 pg/mL in obese individuals; Figure 2), which persisted after including age and sex as a covariate (44.8 ± 13.1 pg/mL in normal-weight; 37.9 ± 12.3 pg/mL in overweight and 38.9 ± 14.0 in obese individuals; *p* = 0.2). 

## 4. Discussion

The main results of the present study suggest that 1,25(OH)_2_D is negatively associated with BMI, LMI and BMD independently of age and sex, whereas no association was found between 1,25(OH)_2_D and FMI in middle-aged sedentary adults.

There is a controversy in the scientific literature regarding the 1,25(OH)_2_D status of obese individuals. It has been described in classical studies that there are greater levels of 1,25(OH)_2_D (~20 to 30%) in both obese men and women [25,26,27,28,46]. However, although we did not find any significant differences between BMI groups, we observed that overweight and obese individuals have ~16.6% and ~15.3% lower 1,25(OH)_2_ than normal-weight individuals, which agrees with a previous large cohort that reported a ~18% lower 1,25(OH)_2_D in the obese group compared with their lean counterparts [23,24]. These discrepancies between studies cannot be attributed to the individual´s age, sex and/or BMI, since the participants had similar biological characteristics. A seasonal effect might explain these controversial results as the 1,25(OH)_2_D assessment was conducted in winter months [46] or spring months [27] in some studies, while others did not control the season in which the blood samples were taken [24]. Given that the effects of ultraviolet ray exposure on 25(OH)D could be different for obese and lean individuals, the time of the year when the study was conducted may be of importance. However, previous studies have suggested that the season of the year does not present the same influence on 1,25(OH)_2_D as it has on 25(OH)D [47]. The different 1,25(OH)_2_D assay methods used across the studies could be one important factor to consider. While older studies applied radioreceptor assays [25,26,27,28,46] in which lipid interferences have been described when samples are not pure enough [48], more recent studies including our own study used modern immuno assays for the assessment of 1,25(OH)_2_D [30,32]. Taken all together, it is likely that obese subjects present low 1,25(OH)_2_D. This fact could be explained because obesity is associated with poor levels of 25(OH)D, and it has been demonstrated that 1,25(OH)_2_D depends on substrate availability [23,49]. A higher fat body mass offers a greater distribution space for both fat-soluble compounds. Additionally, obese individuals are usually exposed to negative lifestyle factors (i.e., unhealthy dietary habits and sedentary behaviour, among others), [50] and to lower sunlight exposure [51]. These lifestyle factors have been shown to have a negative influence on vitamin D status [52,53].

The use of 25(OH)D as a key marker of vitamin D status has logical advantages summarized as greater serum stability and a longer half-life than other markers (e.g., 1,25(OH)_2_D) [22]. However, it is important to consider that it is biologically inactive and therefore could not be the best vitamin D function indicator [54]. The shorter half-life of 1,25(OH)_2_D could be one of the reasons why the relationship of 1,25(OH)_2_D and lean body mass has not been deeply studied. Hassan-Smith et al. reported a positive association between 1,25(OH)_2_D and lean body mass [29]. These findings differ from those observed in our study in which we obtained higher 1,25(OH)_2_D in individuals with lower lean body mass. These discrepancies could be explained by the different biological characteristics of the subjects of the study (i.e., our cohort was older than the Hassan-Smith et al. study [29]) and by the different assay methods used to determine 1,25(OH)_2_D. New studies measuring 1,25(OH)_2_D are necessary to clarify this issue.

We also found a negative association between 1,25(OH)_2_D and BMD in our study cohort which concurs with a previous study that reported a positive association of 1,25(OH)_2_D and the bone resorption marker β-cTX [32]. These results are consistent with the notion that 1,25(OH)_2_D increases bone resorption via stimulating intestinal calcium absorption after calcium intake [32]. Moreover, recent animal and in vitro studies have proposed that 1,25(OH)_2_D has a direct effect on osteoclasts inducing bone resorption by its interaction with the receptor activator of nuclear factor-κβ/receptor activator of nuclear factor-κβ ligand signaling pathway [55,56]. Taking this into consideration, an inverse association would be expected between 1,25(OH)_2_D and bone mineral density. In addition, it seems plausible that 1,25(OH)_2_D is produced when BMD is low and once BMD is recovered 25 (OH)D is transformed into the inactive 24,25(OH)_2_D. Although this argument could explain our findings, further intervention studies are needed to understand this issue.

Our study has some limitations. The cross-sectional design does not allow ascribing causality to the observed relationships. Further, our study population was limited to sedentary healthy middle-aged adults (45–65 years old) and hence these results may not be generalizable to younger, older, and/or physically active individuals. This study, like most clinical studies, was based on a single assay of 1,25(OH)_2_D. We did not assess the 24-hydroxyvitamin D_2_ plasma levels. In addition, a whole-body DXA scan was conducted, so future studies are necessary to investigate whether spine and hip bone mineral density have the same association pattern. Finally, due to the relatively small sample size of the current study, the data should be interpreted with caution. One of the strengths of this study is that body composition was measured using a gold-standard technology, such as dual-energy X-ray absorptiometry. In addition, the measurement of objective physical activity data and dietary intake to be used as covariates represent further strengths.

## 5. Conclusions

In conclusion, our results suggest that 1,25(OH)_2_D could be negatively associated with BMI, LMI and BMD independently of age and sex, while no significant relationship was obtained between 1,25(OH)_2_D and FMI in middle-aged sedentary adults. Intervention studies are needed to understand whether changes in body composition status are associated with changes in 1,25(OH)_2_D in this age-population.

## Figures and Tables

**Table 1:** Descriptive characteristics of participants.

	*N*	All		*N*	Men		*N*	Women	
Age (years)	73	53.7	(5.1)	34	54.6	(5.2)	39	53	(5.0)
**Body composition parameters**									
Body mass index (kg/m^2^)	73	26.7	(3.8)	34	28.3	(3.6)	39	25.3	(3.3) *
Lean mass (kg)	73	43.2	(11.7)	34	53.9	(6.5)	39	34.1	(5.8) *
Lean mass index (kg/m^2^)	73	15.2	(2.9)	34	17.5	(2.0)	39	13.2	(1.8) *
Fat mass (%)	73	40.1	(8.9)	34	34.7	(8.0)	39	44.5	(7.4) *
Fat mass (kg)	73	30.1	(8.5)	34	30.9	(9.8)	39	29.2	(7.1)
Fat mass index (kg/m^2^)	73	10.8	(3.1)	34	10.0	(3.2)	39	11.4	(2.9)
Bone mineral density (g/cm^2^)	73	1.1	(0.1)	34	1.2	(0.1)	39	1.0	(0.1) *
**Dietary intake**									
Total Energy intake (kcal/day)	72	2071.7	(455.4)	34	2312.1	(402.9)	38	1854.6	(390.3) *
Vitamin D intake (µg/day)	72	5.0	(6.0)	34	3.8	(3.3)	38	6.1	(7.6)
Calcium intake (mg/day)	72	763.4	(340.5)	34	867.3	(396.9)	38	670.5	(251.4) *
Phosphorus intake (mg/day)	72	1324.7	(558.9)	34	1507.6	(689.6)	38	1161.0	(342.2) *
**Physical activity parameters**									
LPA (min/day)	70	173.7	(45.4)	33	169.9	(52.7)	37	178.0	(40.7)
MVPA (min/day)	70	95.8	(35.6)	33	96.4	(37.1)	37	96.6	(35.7)
Total PA (min/day)	70	269.5	(75.1)	33	265.2	(79.3)	37	273.3	(72.0)
**Blood parameters**									
1,25 Dihydroxyvitamin D (pg/ml)	73	40.3	(14.1)	34	38.3	(13.4)	39	42.0	(14.6)

Dara are presented as means (standard deviation). Abbreviations: LPA, light physical activity; MVPA, moderate-vigorous physical activity; PA, physical activity. * Significance differences between sexes (*p* < 0.05) obtained by the independent sample T test.

**Table 2:** Association of 1,25-Dihydroxyvitamin D with body mass index, lean mass index, fat mass index and bone mineral density.

	1,25-Dihydroxyvitamin D					
	Model 1		Model 2		Model 3	
	*p* value	β	*p* value	β	*p* value	β
Body mass index (kg/m^2^)	**0.020**	−0.274	**0.040**	−0.263	**0.042**	−0.262
Lean mass index (kg/m^2^)	**0.023**	−0.269	**0.030**	−0.383	**0.032**	−0.383
Fat mass index (kg/m^2^)	0.505	−0.080	0.354	−0.112	0.356	−0.113
Bone mineral density (g/cm^2^)	**0.005**	−0.325	**0.009**	−0.370	**0.009**	−0.377

Model 1 was adjusted for age; Model 2 was adjusted for sex; and Model 3 was adjusted for age and sex. *p* value of multiple-regression analysis. β (standardized regression coefficient). Values in bold indicate significance differences (*p* < 0.05).
